# Paramedic independent prescribing: a qualitative study of early adopters in the UK

**DOI:** 10.29045/14784726.2021.6.6.1.30

**Published:** 2021-05-01

**Authors:** Karen Stenner, Suzanne van Even, Andy Collen

**Affiliations:** University of Surrey; University of West England; South East Coast Ambulance Service NHS Foundation Trust; College of Paramedics; University of Surrey

**Keywords:** advanced clinical practice, non-medical prescribing, paramedic

## Abstract

**Background:**

Paramedics working in advanced practice roles in the UK can now train to prescribe medicine. This is anticipated to benefit patient access to medicines and quality of care where there is a national shortage of doctors, particularly in primary care.

**Aim:**

To explore the experience of paramedics who are early adopters of independent prescribing in a range of healthcare settings in the UK.

**Design and setting:**

A qualitative study involving interviews between May and August 2019, with paramedics in the UK who had completed a prescribing programme.

**Methods:**

Individual interviews with a purposive sample of paramedics recruited via social media and regional paramedic networks. Interviews covered experiences, benefits and challenges of the prescribing role. A framework analysis approach was used to identify key themes.

**Results:**

Participants were 18 advanced paramedics working in primary care, emergency departments, urgent care centres and rapid response units. All participants reported being adequately prepared to prescribe. Key benefits of prescribing included improving service capacity, efficiency and safety, and facilitating advanced clinical roles. Challenges included technological problems, inability to prescribe controlled drugs and managing expectations about the prescribing role. Concerns were raised about support and role expectations, particularly in general practice.

**Conclusion:**

Paramedic prescribing is most successful in settings with a high volume of same-day presentations and urgent and emergency care. It facilitated advanced roles within multidisciplinary teams. Concerns indicate that greater consideration for support infrastructure and workforce planning is required within primary care to ensure paramedics meet the entry criteria for a prescribing role.

## Introduction

With workforce shortages in the NHS (Beech et al., 2019), the number of allied health professionals (AHPs) is expanding in order to help meet demand for general practice, urgent and emergency services (NHS England, 2016, 2017). New models of practice, making use of multidisciplinary teams and evolving advanced clinical practice (ACP) roles, have been developed in response to these challenges (NHS England, 2019). Following a scoping project, AHP prescribing has been extended in the UK, with advanced paramedics being the latest to obtain prescribing responsibilities (NHS England, 2015). Advanced paramedics are defined as experienced autonomous practitioners, educated to Master’s degree level in a subject relevant to their practice and who demonstrate an expert knowledge base, complex decision-making skills, competence and judgement in their area of advanced practice (College of Paramedics, 2018). Core competencies for ACP roles are set out in an agreed multi-professional framework (Health Education England, 2018), with specific competencies agreed for ACPs practising within emergency departments (EDs) (Royal College of Emergency Medicine, 2016) and for paramedic specialists working in primary and urgent care (Health Education England, 2018).

As paramedic roles extend into settings such as urgent treatment centres (UTCs), primary care and hospitals, the scope for prescribing medicines has increased (Evans et al., 2014). Prescribing is intended for paramedics working at advanced level in a role where there is an identified need for the individual to regularly prescribe medicines to patients (College of Paramedics, 2018). The first cohorts undertaking the prescribing qualification post from April 2018 completed and became annotated as prescribers in the summer of 2019. Prior to this, paramedics’ views about prescribing had been generally positive, although concerns about increased stress, poor access to patient records and lack of organisational support were flagged (Bedson & Latter, 2018; Duffy, 2017; Hilton et al., 2019). It is anticipated that paramedic prescribing will yield similar benefits to nurse and pharmacist prescribing, including enhanced patient access to medicines, safety, quality of care and patient satisfaction (Bhanbhro et al., 2011). Previous research on barriers and facilitators to non-medical prescribing (NMP) identified a complex array of personal, financial, education and systems factors, the impact of which varies according to context and setting (Noblet et al., 2017). It is therefore important to evaluate the introduction of prescribing to new professional groups in order to better understand and guide its implementation. At the time of writing, no research had been published on experiences of paramedic prescribing.

### Aim

To explore the experience of advanced paramedics who are early adopters of independent prescribing in a range of healthcare settings in the UK.

## Methods

An interpretive qualitative study using individual interviews with a purposive sample of paramedics who had undertaken the independent prescribing programme in the UK. Recruitment was via a notice posted on paramedic interest groups on social media (Twitter, Facebook and LinkedIn) between May and August 2019. Information sheets were provided 24 hours in advance and informed written consent obtained prior to data collection.

Semi-structured interviews (average 45 minutes long) were conducted by telephone or video call, by an experienced qualitative health researcher (SVE). Topics explored were use of prescribing, benefits and challenges to prescribing and support for the prescribing role (contact author for interview schedule). A pilot interview suggested no changes to the schedule; these data are included in the analysis. Following principles of framework analysis (Ritchie, 2003), a thematic framework of known benefits and challenges to NMP was developed, against which coded transcripts were indexed. ATLAS.ti (Version 5.5) software was used to aid coding and data analysis. Conceptual saturation occurred, with no new categories arising after interview 15. Data were then mapped and interpreted using a reflexive process and the framework adapted as new conceptual areas were chartered (Pope & Mays, 2006). Themes were discussed and agreed within the research team which included experienced qualitative health researchers (KS, SVE) with expertise in NMP research (KS) and a consultant paramedic (AC). Interviews were recorded using an external password-protected audio-recording device and transcribed verbatim; transcripts were checked for accuracy and anonymised.

## Results

### Participant profile

A total of 18 paramedic prescribers were interviewed between July and August 2019, of which seven were prescribing and 11 had passed the prescribing qualification and were awaiting annotation. Table 1 shows participant demographics.

**Table 1.** Participant demographics.

	N (total = 18)	%
**Age** (mean 41, range 28–53)		
20–29	1	5.5
30–39	6	33.3
40–49	8	44.4
50–59	3	16.6
**Sex**		
Male	14	77.7
Female	4	22.2
**Job title**		
Advanced practitioner or advanced paramedic practitioner	7	38.8
Advanced clinical practitioner (or trainee)	5	27.7
Rapid response practitioner	1	5.5
Other advanced role (advanced critical care practitioner, paediatric practitioner, emergency care practitioner)	3	16.6
Senior practitioner, clinical lead	2	11.1
**Number of years as a paramedic** (mean 14.5, range 5–25)		
Up to 9	5	27.7
10–19	10	55.5
20–29	3	16.6
**Number of years at advanced-level practice**		
0–4	9	50
5–9	5	27.7
10–14	4	22.2
**Qualification at advanced practice**		
Undertaking doctorate	1	5.5
Master’s in advanced practice	7	38.8
Undertaking Master’s in advanced practice	5	27.7
Postgraduate diploma/modules	4	22.2
Bachelor’s in advanced practice	1	5.5

### Setting and scope of prescribing practice

Participants worked in primary care (n = 10), out-of-hospital (n = 4) and in-hospital (n = 7). Three participants worked across more than one setting (counted twice in the above), including rotational models incorporating work in primary care and the ambulance service. Primary care roles typically covered urgent or same-day appointments, out-of-hours, house visits and telephone consultations. Two participants worked in UTCs. Pre-hospital work included ambulance, air ambulance and community rapid response. Secondary care roles included intensive care units (ICUs) and EDs, including specialist paediatric emergency.

A broad scope of practice was described, covering acute presentations of minor illness, injury and/or accident and emergency. Where a personal formulary (list of medications to be prescribed) had been agreed, this was mainly set out by medication groups rather than specific medications.

In general practice, paramedics were primarily involved with same-day appointments, treating patients with acute presentations (e.g. infections) or exacerbations of long-term conditions, such as asthma. Patients seeking ongoing care for chronic conditions were typically routed to a GP. Typical medication groups covered in primary care included antibiotics, anti-inflammatories, painkillers, inhalers, creams and steroids. A minority (more experienced) prescribed anti-depressants. The majority prescribed around 10 prescriptions per day, although two participants estimated 20–40 prescriptions per day. Those working in UTCs reported prescribing 5–10 prescriptions per day.

In secondary care, participants prescribed analgesia, anti-seizure medications, cardiovascular drugs, steroids, emergency drugs (e.g. for resuscitation) and fluids, but also pain relief, antibiotics and allergy medications. Conditions most commonly treated included trauma, cardiovascular disease, seizures, COPD, asthma and stomach problems. Prescribing rates ranged from eight to over 50 prescriptions a day in ICUs.

### Key benefits

Benefits are detailed in Table 2.

**Table 2.** Categories of benefits and barriers and the number of participants reporting each category.

Benefits	N
**Patient care**	
Faster access to medicine for patients / reduced treatment delay	12
Reduced waiting time for patient	12
Improved quality of care, optimised care	12
Complete episode of care in single appointment	9
Safer / more appropriate prescribing	8
Improved communication about treatment and medicines	7
Reduced length of stay / admission to hospital/A&E	5
Improved patient satisfaction, confidence or trust	3
Improved continuity of care	3
**Healthcare systems and teams**	
Reduced ‘door hanging’ (time spent arranging for someone else to prescribe patient’s medicine)	15
Replaces appointment with doctors and allows doctors more time for complex cases	11
Improved service efficiency – meeting more appointments, better flow	11
Expanded service reach, capacity or range or hours covered	9
Reduced prescribing costs due to less inappropriate prescribing	6
Improved team work, upskilling and integration	5
**Personal and professional**	
Facilitates career development and opportunity	12
Improved job satisfaction	12
Increased professional recognition or respect	11
Alignment of responsibility with other ACPs – essential to role	7
Increased knowledge and ability for safe and appropriate prescribing	7
Barriers and concerns	N
**Systems/national level**	
Inability to prescribe CDs	15
IT prescribing support system problems	6
Poor alignment of prescribing regulations across different ACPs	3
Poor access to prescribing budget or medical records in community	2
**Individual/personal**	
Increased anxiety and stress	12
Concern about error and level of risk and responsibility	10
Concern about increased workload due to prescribing	1
**Interpersonal**	
Misunderstanding of the prescribing role and its boundaries – pressure to prescribe out of scope of practice	11
Managing patient expectation to prescribe (inappropriately) antibiotics, medicine available to purchase in pharmacies	5
Isolation and access to support in out-of-hours/community	2
**Wider concerns**	
Concern over adherence to regulation that prevents under-experienced paramedics from prescribing within poorly supported areas of practice (and the potential impact on patient safety and professional reputation)	11
Concern over retention of paramedics in the ambulance service or pressure to become advanced paramedic	7

ACP = advanced clinical practice; CD = controlled drugs.

#### Patient care and healthcare systems

Advanced paramedics with prescribing responsibility were reported to increase the capacity of multidisciplinary teams to deliver primary, urgent, pre-hospital and secondary care, relieving workforce shortages and contributing to team knowledge and expertise. By providing urgent care clinics, cover for out-of-hours and house calls, participants considerably increased capacity and facilitated doctorless services. Key benefits were improved speed and efficiency of services and reduced waiting time (for patients and staff), freeing doctor time for more complex cases.

*I think within the emergency department the biggest benefit is the fact that you can work entirely independently and you can keep that patient flow, because any time that you have to go and communicate with another person in order to give them the information that they would need to be able to prescribe, is diverting them away from what they were doing and potentially it’s introducing a source of error.* (9, ACP, ED)

Independent prescribing was said to facilitate appropriate choice of medicine, improving the quality of care and enhancing the level of advice and information given to patients.

*It enables me to give a wider range of treatments to the patients, to actually treat them with the best choice of drug rather than a drug that is allowed, as such, from a paramedic’s standpoint. It also allows me to give more timely treatment. […] So, from a patient point of view, they get seen quicker, they get the treatment quicker, they get home quicker, or referred to speciality quicker.* (7, ACP, A&E)

*You get a lot of people who come in who have seen somebody else, for instance, who are not sure or haven’t fully understood how to take the medicine that’s already been prescribed, or are concerned about interactions.* (5, advanced practitioner, primary care)

#### Personal and professional

Prescribing, described as a requirement for many ACP roles, was considered essential to be able to conduct these roles safely and efficiently. Motivation for becoming a prescriber included individual role progression, learning and improving patient care. Having a prescribing qualification was said to increase career opportunities, putting paramedics on a more equal footing with other ACP professionals, such as nurses or pharmacists. Many believed that prescribing brought recognition and respect to the paramedic profession and improved job satisfaction, as well as increasing knowledge and confidence around pharmacology.

*It’s wonderful to have a profession that’s only been a profession for less than 20 years actually be given the recognition that we are competent independent clinicians and can make these sorts of level of decisions and actually take that care forward, and also expand the avenues down which paramedics can go.* (7, ACP, A&E)

*I know more than I did before, and I also know more about my limitations, and I also know just to be extra careful and maybe be a bit more aware of some of the pitfalls regarding polypharmacy and just making sure you’re prescribing safely and effectively and passing on that safety net advice to the patient and making sure that they understand what the medicines are for and improving the health literacy of that.* (10, trainee advanced practitioner, emergency and primary care)

### Key challenges

Key challenges are detailed in Table 2.

#### Legislation to prescribe controlled drugs

Participants with longer experience in primary care, out-of-hours or house calls or with an extended remit to provide end-of-life or palliative care reported a need to prescribe controlled drugs (CDs).

*That is going to be quite a limitation at the moment. It impacts quite a broad range of patients. It is not insurmountable, but it is a pain. As paramedics where we are used to using controlled drugs in our ambulance practice, it feels particularly limiting.* (16, advanced paramedic, primary care)

Considered integral to specialist roles in secondary care and EDs, the inability to independently prescribe CDs was limiting, and restrictions applied to different professions caused confusion. There was concern that employers may favour nurse and pharmacist ACPs who can prescribe a range of CDs.

*A lot of the prescribing that we need is obviously opiate based pain relief.* (14, consultant ACP, ED)

*You have got to really think about can I give that medication? … Everybody has got to explain their full job role, you know, all these weird intricacies when somebody actually just wants to know: ‘Can you do what is the equivalent of this job?’.* (3, trainee ACP, ICU)

#### Administrative IT issues

Teething problems with electronic prescribing systems, which at the time of writing did not recognise prescriber registration numbers, caused frustration. Lack of access to patient medical records and/or prescribing budget restricted the ability to prescribe in out-of-hours and pre-hospital settings.

*We had to add a zero to my HCPC number so that it recognised the number.* (4, paramedic practitioner, primary care)

#### Managing expectations about the prescribing role

The need to manage colleagues’ expectations of paramedic prescribing responsibilities was commonly mentioned. Participants were engaged in boundary setting around ‘third party’ prescribing, which is when prescribers are asked to issue repeat medicines or prescribe for patients they have not assessed.

*The number one thing on my mind is that I’m recognised as a senior member of the team. I’m already a go-to person for discussing patient care and pathways. The risk is that people think, ‘Oh, he can just write my prescription now’, but in my mind, apart from what I’ve just told you about I’ve got a lot of experience, I still am very new as a prescriber, and what I’m conscious of is stepping outside of my fledgling prescribing skills, if you like, just to match my current role. So, I guess I keep telling everyone, ‘Don’t start coming to me [laughter] and ask me to write repeat prescriptions’.* (15, clinical lead, UTC)

Managing patient expectations mostly involved problems common to all prescribers, such as managing demand for inappropriate antibiotics. However, there were wider concerns about the impact of advanced paramedic roles on retention of paramedics within the ambulance services and pre-hospital care. While rotational roles and portfolio careers were considered advantageous for developing a range of experiences in preparation for advanced roles, the need to value core pre-hospital skills was stressed.

*I think it is really important to have paramedics that are still going to be utilised for accident and emergency. So if you have a problem that you need to call 999 then it is really important that there are still going to be paramedics to fulfil that role.* (17, advanced paramedic, primary and emergency care)

### Support and preparation

#### Prescribing programme

Most participants felt the prescribing course and days of supervised practice had prepared them with the necessary knowledge and confidence to prescribe. However, more information was requested on legislative issues specific to paramedic prescribing. Time spent with a designated medical practitioner in a relevant area of practice was considered invaluable in building competence and confidence. Some reported difficulty with matching time for supervision.

#### Preparation for advanced role

Advanced-level experience and training were considered essential for managing long-term conditions, mental health and prescribing for neonates, children and pregnant women. The ACP framework for EDs (16) was regarded as a facilitator in preparing scope of practice in EDs; however, preparation for primary care roles appeared patchy and less well-defined. There was a concern that patient safety and the reputation of the profession could be at stake if novice paramedic prescribers were recruited into ACP roles without a period of training and experience. Concerns were also expressed around ‘scope creep’ – that is, being asked to take on more responsibility and the potential for workload and stress to increase in these roles over time.

*I think there’ll be a bigger expectation that our workload will increase. I think more will be expected of us … ‘Well all these paramedics are prescribing so this will make my life easier’. We’ll be put upon a lot more, if you know what I mean.* (8, senior practitioner, UTC)

#### Anxiety and the importance of a supportive environment

Anxiety was expressed in relation to inadvertently causing harm to a patient, being involved in a dispute or litigation and potential negative impact on the profession. While anxiety was expected to dissipate with experience, more enduring concerns were expressed about litigation and the consequences of making a medication error due to prescribing for patients with multi-morbidity, within a high pressure, time-precious work environment.

*I think the main challenges are just that it’s a big responsibility and you’re seeing patients who are on lots of medications, they might have underlying medical conditions, and you’re aware of all the interactions that can happen with medications and the side effects, so the pressure is to make sure you do that safely and effectively.* (10, paramedic practitioner, rotational model GP setting)

Having a supportive working environment to help mitigate against isolation, safety concerns and related anxiety was considered important by all. Paramedics both contributed to and benefited from the range of skills, expertise and mutual support within multidisciplinary teams. There were significant concerns about paramedics prescribing in isolation of team support, such as in the ambulance service.

*Paramedics will be dealing with the most unwell patients, with comorbidities, complex, multi-pharmacy, polypharmacy, so it’s imperative that they work as part of a team to understand that or it may be detrimental to the patient. That’s why the idea of paramedic independent prescribing is for paramedics to work as part of multidiscipline teams. It’s not for the ambulance services.* (11, advanced paramedic, primary care)

## Discussion

Advanced paramedic prescribing is rolling out in line with expectations with similar benefits/barriers to those reported by other NMPs (Graham-Clarke et al., 2018; Noblet et al., 2017). Paramedic skills and experience can benefit multidisciplinary team working, and improve efficiency, capacity and quality of care. Independent prescribing facilitated the expansion of new and innovative roles for paramedics, as reported by other professions (Carey et al., 2017; Hindi et al., 2019), and was considered an essential component for many advanced roles. Furthermore, it facilitated parity of access to ACP roles and this was a strong motivator to undertake the prescribing qualification. Participants felt sufficiently prepared to prescribe, although more clarity was requested over legal differences between professions as taught in prescribing programmes, to avoid misunderstanding of scope of prescribing practice.

In line with the ‘diffusion of innovations’ theory (Rogers, 2003), adoption of NMP can be seen to occur in stages, as well as enacting change at the national, organisational and individual level. Early barriers (such as IT issues) may be temporary. Planned changes to the Misuse of Drugs Acts allowing independent prescribing of a limited range of CDs by paramedics will take time to implement and may require future revision, as occurred with nurse prescribing (Stenner & Courtenay, 2007). More enduring concerns are poor access to patient records in community settings, the need for clinical support and clear professional role boundaries – barriers known to restrict optimal use of prescribing (Courtenay et al., 2011, 2012). Participants cautioned against paramedics prescribing in unsupported and isolated roles; a concern echoed by other professions in primary care (Hindi et al., 2019). Equally, prescribing was not considered appropriate within the ambulance service where access to a doctor and patient records is poor, echoing doubts about the necessity of prescribing within rotational paramedic models for similar reasons (Turner & Williams, 2018).

While ACP competencies for emergency care (Royal College of Emergency Medicine, 2016) provide an agreed framework for role development, advanced roles in primary care appear to be shaped by individual preferences and local needs and require careful management of expectations and role boundaries (Abrams et al., 2020; Nelson et al., 2019). Clear credentialing for advanced paramedic prescribers in primary care is recommended, along with use of clinical governance tools (Pharmacists’ Defence Association, 2019) to clarify scope of practice and reduce role misunderstanding. Support for managing the transition to primary care with its associated increased uncertainty and responsibility is an identified challenge for advanced practitioners (Nelson et al., 2019). It is therefore important to reiterate that prescribing responsibilities should be restricted to those with appropriate cred entials, working at advanced level and receiving the necessary clinical support.

### Limitations

As a small study, generalisation is limited and findings may change as paramedics become experienced prescribers. A repeat study is recommended to assess whether barriers identified in this study have been addressed. There is scope to further assess economic impact and to compare outcomes across the various settings in which paramedics prescribe.

## Conclusions

Advanced paramedic prescribers can increase capacity to treat same-day presentations in urgent and emergency care settings and where there is a high volume of low complexity patients requiring prescriptions in primary care. This study identified concerns about burnout and stress when prescribing within a high-pressured environment for patients with complex needs, a finding that echoes experiences of GPs (Cheshire et al., 2017). It is questionable to what extent advanced paramedics can alleviate workforce shortages over the long term unless underlying causes of low recruitment and retention in healthcare are addressed. However, with the right level of support, extending the skills mix within multidisciplinary teams may accelerate mutual learning and support, creating a more sustainable model for future healthcare.

## Acknowledgements

The authors would like to thank participants for their time and engagement with this research.

## Author contributions

KS was responsible for the study conception and design, contribution to checking transcripts, coding, analysis and drafting and amending the article. SVE was responsible for data collection, transcription, coding, analysis and critical review of the submission. AC contributed to design, analyses and interpretation of findings as well as critical review of the submission. KS acts as the guarantor for this article.

## Conflict of interest

None declared.

## Ethics

The project was assessed via the University of Surrey Ethics Committee self-assessment process.

## Funding

This research was funded by the University of Surrey Faculty of Health and Medical Sciences Research Support Fund.
